# Posterior mini-incision total hip arthroplasty controls the extent of post-operative formation of heterotopic ossification

**DOI:** 10.1007/s00590-015-1646-x

**Published:** 2015-05-08

**Authors:** D. S. Edwards, S. A. R. Barbur, A. M. J. Bull, G. J. Stranks

**Affiliations:** Royal Centre for Defence Medicine, Birmingham, UK; The Royal British Legion Centre for Blast Injury Studies, Imperial College London, South Kensington, London, SW7 2AZ UK; Hampshire Hospitals NHS Trust, Basingstoke, Hampshire RG24 9NA UK

**Keywords:** Heterotopic ossification, Arthroplasty, Hip, Mini-incision, Posterior approach

## Abstract

Heterotopic ossification (HO) is the formation of bone at extra-skeletal sites. Reported rates of HO after hip arthroplasty range from 8 to 90 %; however, it is only severe cases that cause problems clinically, such as joint stiffness. The effects of surgical-related controllable intra-operative risk factors for the formation of HO were investigated. Data examined included gender, age of patient, fat depth, length of operation, incision length, prosthetic fixation method, the use of pulsed lavage and canal brush, and component size and material. All cases were performed by the same surgeon using the posterior approach. A total of 510 cases of hip arthroplasty were included, with an overall rate of HO of 10.2 %. Longer-lasting operations resulted in higher grades of HO (*p* = 0.047). Incisions >10 cm resulted in more widespread HO formation (*p* = 0.021). No further correlations were seen between HO formation and fat depth, blood loss, instrumentation, fixation methods or prosthesis material. The mini-incision approach is comparable to the standard approach in the aetiology of HO formation, and whilst the rate of HO may not be controllable, a posterior mini-incision approach can limit its extent.

## Introduction

Heterotopic ossification (HO) is the formation of ectopic bone at extra-skeletal sites [1]. In the case of total hip arthroplasty (THA), it is commonly found in the periarticular capsule and surrounding muscular structures [2]. The rates of HO after hip arthroplasty have been reported to range from 8 to 90 % [3, 4]. Brooker et al. [5] have discussed the risk factors for HO formation and developed a method of quantitative classification. It has been shown that grades 3 and 4, the final stage of ankylosis, are the most problematic for patients, commonly complaining of pain and a reduced range of movement [6]. Rates of severe HO range from 3 to 55 % depending on patients and surgical factors present [7]. Established risk factors include male gender, diffuse idiopathic skeletal hyperostosis (DISH), ankylosing spondylitis, previous THA and subsequent HO formation, rheumatoid arthritis, osteonecrosis and previous trauma to the hip [8, 9]. However, there exists little or weak evidence pertaining to the controllable intra-operative factors such as incision length, blood loss, length of operation, prosthesis type and instrumentation [3–5, 7, 8, 10].

Recent research investigating the effect of inflammation and local ischaemia on stem cell manipulation and subsequent HO formation has proposed the possibility that HO formation is likely to be stimulated by local and systemic factors contributing to the pathological picture [1, 11–16]. In the case of THA, it is surgery itself that indicates the index event leading to HO. Therefore, the purpose of this study was to test the hypothesis that decisions made by the surgeon can influence HO formation following THA. This was done by identifying a large patient cohort, quantifying the current incidence and report on surgical and intra-operative factors that may predispose to the formation of HO. Our hypothesis is that factors that influence HO rates are surgical technique, including incision length, a measure of tissue trauma and intra-operative measures such as prosthetic fixation type, the use of pulsed lavage and canal brush, component size and material, and relevant patient variables, for example age, gender and fat depth.

## Methods

**Fig. 1:**
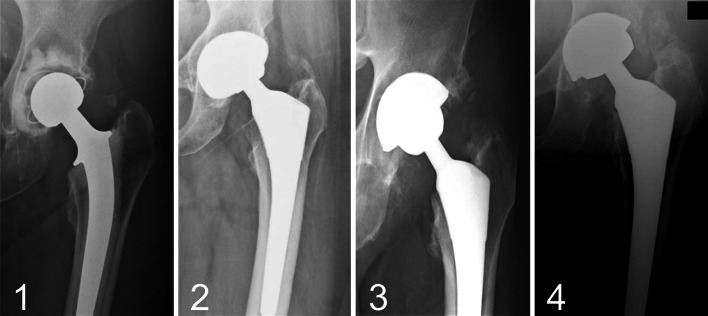
Brooker’s classification (classes *1*–*4*) of HO after hip arthroplasty

An internal single hospital database was used, and consecutive THA patients performed by a single arthroplasty surgeon were retrospectively evaluated. Inclusion criteria for the study were as follows: a complete follow-up at 1 year with available radiographs of the operated hip, no prophylaxis of HO given and the availability of data (in the database or patient case notes or combined). Revision THA, THA for congenital hip disorders, trauma-related conditions and THA associated with osteotomy were excluded. Post-operative radiographs taken at 1 year were evaluated for the presence of HO and quantitatively scored as per Brooker’s [5] classification (Table 1; Fig. 1). The classification of Schmidt and Hackenbrock [17] was used to denote the anatomical site of the HO (Table 2). Table 3 lists the database variables analysed.

**Table 1:** Brooker’s classification of heterotopic ossification of the hip joint [5]

Score	Specification/region
0	No HO on anteroposterior view of the hip
Class 1	Islands of bone within the soft tissues about the hip
Class 2	Bone spurs from the pelvis or femur with >1 cm between opposing ends
Class 3	Bone spurs with reduced space between the opposing bone surface of <1 cm
Class 4	Ankylosis of the hip joint

**Table 2:** Anatomical classification of HO from Schmidt and Hackenbrock [16]

Score	Specification/region
0	No HO on anteroposterior and standard lateral view of the hip
I	HO strictly below the tip of the greater trochanter
II	HO below and above the tip of the greater trochanter
III	HO strictly above the tip of the greater trochanter

**Table 3:** Surgery-related variables evaluated

Gender	Age at operation
Femoral stem fixation method	Femoral head material
Femoral head size	Anaesthetic type
Use of pulsed lavage	Use of femoral canal brush
Anti-thrombosis therapy	Length of operation (min)
Blood loss (cc)	Incision length at start (cm)
Incision length at end (cm)	Fat depth (cm)
Incision length group (group I >10 cm, group II ≤10 cm)	

Data were assessed for normality using the Shapiro–Wilk test and the alpha value set for significance set at *p* < 0.05. The study was registered with the local hospital trust Audit and Research Department. Gender, femur fixation method, femoral head size, acetabular diameter, the use of pulse lavage, blood loss, age, prosthesis material, anaesthetic type, operative time, fat depth and incision length were evaluated for HO formation (present/not present), grade (Brooker) and extent (Schmidt). The Chi-squared test was used for categorical data, *t* test for comparison of means between the HO and non-HO groups for numerical data and Pearson’s correlation to demonstrate linear relationship between groups. Where significance was found in scalar data, Chi-squared test was used to determine significance from the mean.

## Results

**Fig. 2:**
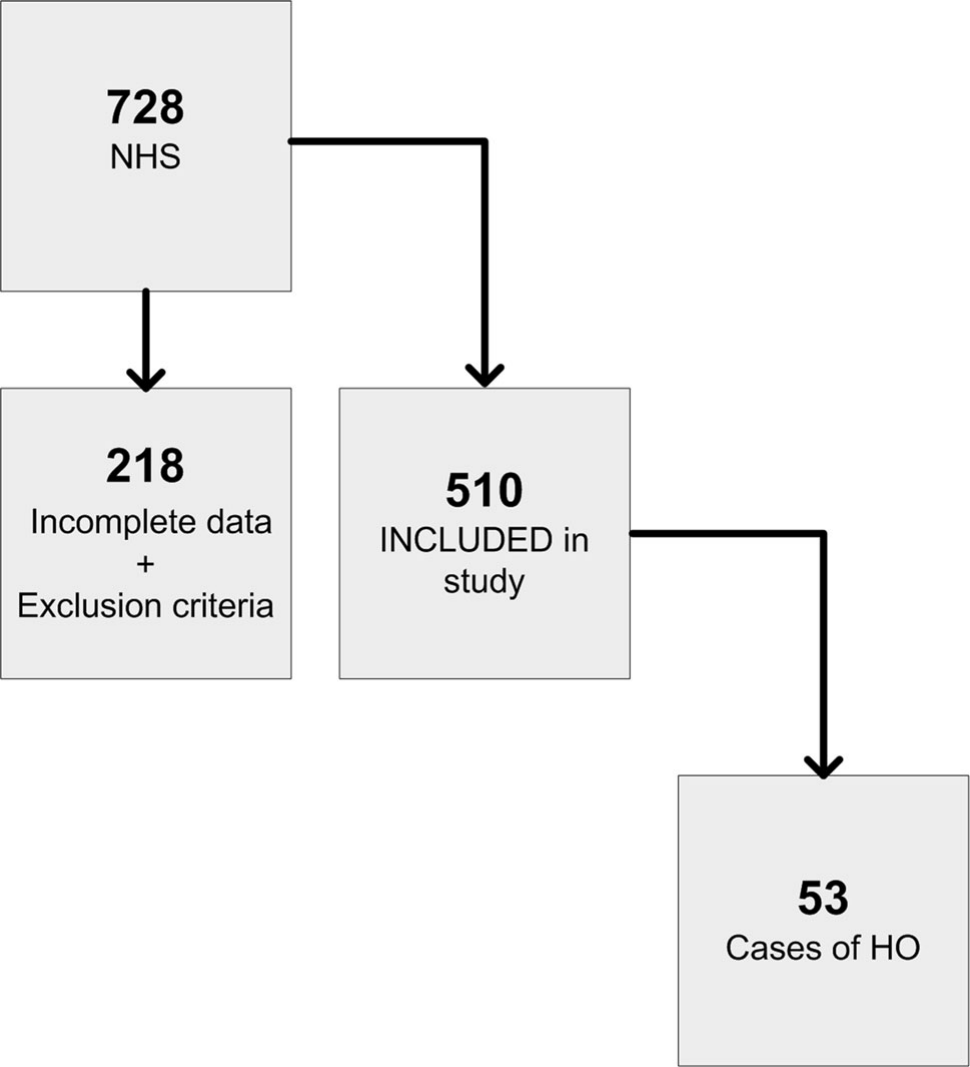
A flow diagram of patient inclusion

Post-exclusion, 510 cases were available for a 15-year period (Fig. 2). All cases included were performed by the same surgeon of Consultant grade (senior author—GJS) using the posterior approach to the hip. The average age of the cohort was 65.8 years (range 21.8–93.5 years, standard deviation 11.65 years). There were 221 (43.3 %) males and 289 females (56.6 %). Fifty-three patients (10.4 %) demonstrated evidence of HO at the 1-year follow-up radiograph with one demonstrating a Brooker grade 4, complete ankylosis (Table 4). The fixation method of the prostheses in 389 cases was uncemented (76.3 %) and 121 cases (22.7 %) cemented. Cobalt chrome was the most common femoral head material (321; 62.9 %) with ceramic accounting for the remaining 189 (37.1 %). The Biomet Biometric-M2A(metal)/Exceed ABT(ceramic) was the commonest combination of prosthetic design (65.1 %). Surgical variables measured are summarised in Tables 5 and 6.

**Table 4:** HO cases classified into Brooker grade

Brooker grade	Frequency	% of overall cohort
1	40	8.1
2	5	1.0
3	7	1.4
4	1	0.2
Total	53	10.7

**Table 5:** Categorical data statistics

Variable	Total	HO present	HO not present	*p* value
Gender				
Male	221	30	191	0.027*
Female	289	23	266	
Femur fixation				
Cemented	121	15	106	0.117
Uncemented	389	38	351	
Femoral head material				
Metal	321	35	286	0.450
Ceramic	189	19	170	
Anaesthetic type				
General anaesthesia	344	36	308	0.316
Spinal anaesthesia	142	17	125	
Epidural anaesthesia	24	0	24	
Pulsed lavage				
Yes	90	13	77	0.084
No	420	40	380	

* Significant at *p* < 0.05

**Table 6:** Scalar data statistics

Variable	Value	Correlation with HO grade (Brooker)	Correlation with HO extent (Schmidt)
Age (years)			
Mean	65.81	0.558	0.207
Range	21.8–93.95		
SD	11.65		
Blood loss (ml)			
Median	300	0.523	0.208
Range	0–7000		
SD	202		
Operative time (min)			
Median	60	0.047*	0.990
Range	35–200		
SD	15.68		
Fat depth (cm)			
Median	3	0.902	0.149
Range	0–12		
SD	1.59		
Incision length (cm)			
Median	10	0.620	0.007*
Range	7–30		
SD	3.45		
Femoral head size (mm)			
Median	38	0.912	0.495
Range	25–56		
SD	4.15		
Acetabular diameter (mm)			
Median	52	0.817	0.490
Range	28–66		
SD	4.0		

* Significant at *p* < 0.05

Male patients were statistically more likely to develop HO (*p* = 0.027). Longer operations, >60 min, caused higher grades of HO (*p* = 0.047). Incision length was positively correlated with the extent of HO. HO was limited to areas above the greater trochanter (Schmitt grade 3) in smaller incisions (*p* = 0.007). Incisions >10 cm resulted in more widespread HO formation (*p* = 0.021). No other significant results were found, including prosthetic design or material.

## Discussion

This study has shown that smaller incisions limit the extent of HO that appears within 1 year post-total hip arthroplasty, using the posterior approach, in a case series of 510 patients. The overall rate of HO was 10.4 %. In addition, longer operations increased the severity of HO and male gender was associated with higher rates of HO.

The HO incidence in this study of 10.4 % is comparable with the lower end of previous published ranges, 8–90 % [3, 4]. The number of THAs performed in the UK in 2013 was over 80,000 [18] and is expected to rise year on year with an ageing population with increased mobility demands. The number of HO-affected hips following THA will also subsequently rise. Literature demonstrates a significant reduction in range of motion gain due to arthroplasty seen in patients who develop Brooker grades 3 and 4 HO [6]. As 15 % of our HO cohort developed these grades, primary prevention, prophylaxis or limiting the extent of HO is therefore necessary. Anti-inflammatory medication and radiation therapy have been proven to reduce the formation and recurrence in high-risk groups [19–22]. However, both modalities may be contraindicated in an ageing patient. Research demonstrates that pro-inflammatory chemokines and cytokines are responsible for induction of osteoblast activity [11–13, 23]. Inflammation and tissue damage/ischaemia are therefore likely to be the key in the formation of HO.

Gender [24], comorbidities [8], genetic susceptibility [25, 26] and HO formation with previous surgery are known risk factors for HO formation; these are beyond the surgeon’s control. Furthermore, concerns exist that localised causes of ischaemia and increased lactate levels render tissues to an increased risk of HO [16]. We explored surgery-related factors and intra-operative variables that can be controlled which may contribute to the inflammatory response amplitude. Incision length, tissue dissection and subsequent localised trauma and ischaemia, blood loss, anaesthetic type and length of surgery may all contribute to the local inflammatory response. Pulsed lavage may also spread osteoblast precursors, thereby creating an osteoconductive environment. It could be argued that mini-incision THA either reduces the “zone of injury” to the skin and underlying soft tissues and subsequent risk of HO formation or increases the risk due to increased tension on the wounds and soft tissues by the retractors. This work provides evidence for the former, but not the latter. Whilst 10 cm is regarded as the cut-off between standard and mini-incision, it is the deeper muscle and soft dissection that is most relevant for the formation of HO [27].

The main limitation with this study is that this is retrospective cohort data. In the comparison of groups where the incision is greater than the median of 10 cm, the incision is occasionally enlarged due to operative necessity and not surgeon’s choice. However, inter-operative variables are minimised by our single-surgeon cohort.

Our results demonstrate that differences in intra-operative practice do not contribute significantly to the factors that initiate HO formation. It is therefore an “all or nothing” event where the act of surgery itself, no matter how extensive, is the contributing factor. However, the data suggest that the surgeon may control the extent and nature of HO formation by limiting the incision length and if possible the length of the operation. This may be advantageous in trying to reduce the numbers of patients affected by the higher grades of HO and subsequently loss of range of motion. Our findings regarding the relationship between gender and HO formation mirror that which has been previously reported. Minimal invasive surgery, fixation method, prosthetic material, femoral head size and material do not correlate with HO formation.

## Conclusion

An HO rate of 10.2 % in our cohort, where minimal incisions are used (median 10 cm), is towards the lower end of the range of published rates of HO. We therefore conclude that the posterior minimal incision is a safe approach to use with regard to the formation of HO. Furthermore, the minimal incision technique reduces the extent of HO found in the soft tissue.
